# Correction: After the Crimea crisis: Employee discrimination in Russia and Ukraine

**DOI:** 10.1371/journal.pone.0244659

**Published:** 2020-12-23

**Authors:** Iuliia Naidenova, Cornel Nesseler, Petr Parshakov, Aleksei Chusovliankin

The following information is missing from the Funding statement: This paper is an output of a research project implemented as part of the Basic Research Program at the National Research University Higher School of Economics (NRU HSE).

There is an error in affiliation 1 for authors Iuliia Naidenova, Petr Parshakov, and Aleksei Chusovliankin. The correct affiliation 1 is: HSE University, Perm, Russia.
