# Supplementary material for: After the Crimea crisis: Employee discrimination in Russia and Ukraine
Source: PLoS One. 2020 Oct 28;15(10):e0240811. doi: 10.1371/journal.pone.0240811 (PMC7592748; doi:10.1371/journal.pone.0240811)
Supplement: S1 Table — (PDF) [file pone.0240811.s001.pdf]

S1 **Table.**
Russian and Ukrainian soccer – distance to Crimea excluded.

	Dependent variable: log(average minutes)			
Model	(1)	(2)	(3)	(4)
League country	Russia	Ukraine	Russia	Ukraine
Control group	Foreigners	Foreigners	All players	All players
Ukrainian after crisis	-0.620*		-0.535	
	(0.331)		(0.348)	
Russian after crisis		-0.572*		-0.527
		(0.299)		(0.315)
Ukrainian player	0.152		0.153	
	(0.182)		(0.202)	
Russian player		-0.227		-0.214
		(0.147)		(0.151)
After crisis	-0.018	0.107	0.011	0.261**
	(0.151)	(0.177)	(0.113)	(0.123)
Market value in euro	0.062***	0.106***	0.079***	0.102***
	(0.008)	(0.013)	(0.011)	(0.018)
Zero market value	-1.239***	-0.471**	-1.030***	-1.173***
	(0.261)	(0.225)	(0.152)	(0.106)
Age	0.218**	0.424***	0.595***	0.326***
	(0.095)	(0.138)	(0.085)	(0.068)
Age sq.	-0.003*	-0.007**	-0.010***	-0.005***
	(0.002)	(0.003)	(0.002)	(0.001)
Participated all games	0.745***	0.745***	0.922***	0.938***
	(0.188)	(0.086)	(0.125)	(0.087)
Team change during season	0.142	0.000	-0.125	0.084
	(0.120)	(0.127)	(0.094)	(0.074)
Player position effects	Included	Included	Included	Included
Club effects	Included	Included	Included	Included
Season effects	Included	Included	Included	Included
Club position	-0.060**	-0.019	-0.030	0.030**
	(0.027)	(0.014)	(0.022)	(0.011)
Constant	0.269	-4.195**	-4.873***	-2.118**
	(1.312)	(1.829)	(1.153)	(0.937)
Observations	1,268	811	2,935	2,770
R-squared	0.201	0.198	0.252	0.268

Robust standard errors in parentheses. *** p<0.01, ** p<0.05, * p<0.1
