# Supplementary material for: After the Crimea crisis: Employee discrimination in Russia and Ukraine
Source: PLoS One. 2020 Oct 28;15(10):e0240811. doi: 10.1371/journal.pone.0240811 (PMC7592748; doi:10.1371/journal.pone.0240811)
Supplement: Attachment — Submitted filename: Reviewer.docx [file pone.0240811.s002.docx]

**Reviewer #1:**

the topic that you address in your paper is certainly important. I like the idea to use a political shock to identify changes in employment outcomes and I particularly like the idea to use sports data. However, I have a couple of major and minor points that need to be addressed before the paper can be considered suitable for publication.

Dear reviewer, thank you for the time and effort that you put into improving this paper. We highly appreciate it. We tried our best to incorporate all the changes, also in accordance with the demands from the other reviewer. Please find detailed answers to the specific comments below:

First, I have no idea how you arrive at your interpretation of the coefficients. My reading of the tables suggests that none of the relevant coefficients is statistically significant. What do the result tables contain? Is it the standard errors of the coefficients or the t-values? Did you cluster the standard errors by year/season? Since time elapsed may play a role, this should be done.

We have changed the table to mark the significance of the coefficients. We cluster standard errors over the clubs, and control for the years in the regression. We agree that time matters in term of standard errors, but since we can’t cluster over season and club we decided to cluster over the club. We believe that it is necessary to account for club-specific variation. We have added this discussion to the text of the paper (p.4).

Second, yellow cards in soccer can be the result of either player or referee behavior. Since you cannot distinguish between the two sources, this test cannot produce meanungful results (are players more aggressive after the annexion or do referees punish players from the other country more harshly?)

We agree with the referee that we cannot distinguish if either players and/or referees behave differently. We explicitly mention, “We use number of yellow cards to understand if players behave more emotionally after a political crisis or if referees negatively discriminate them.” (p. 10)

On page 1 we write, “From the Russian government’s perspective the annexation was a natural incident or natural process that was bound to happen.” It is important to stress that the Russian government’s perspective is that any behavioral change (independent of the perpetrator being Russian or Ukrainian) in Russia would run counter to this argument. Thus, while we would prefer to know if either player or referee is responsible for the behavioral change, this examination is nonetheless interesting.

We understand that we did not explain this part of the paper carefully enough. Therefore, we add an explanation.

Third, the number of observations in your descriptives tabels is far larger than the number of observations in the estimations. Why do so many player-season-observations disappear?

We have updated the tables with summary statistics. For some regressions the number of observations is lower than in the summary table because, we exclude domestic players in order to have a control group of foreigners only.

Fourth, you state that some Ukrainian hockey teams disappeared after the annexion. Are these teams that previously employed a large or a low number of Russian players? Is there any selection going on with the disappearing teams?

Yes, some clubs disappear. There were nine clubs before the Crimea crisis and eight after. So, the number of clubs is almost the same, but some of them, changed their location. The share of Russian players is different for the clubs which remain in the league compared to those clubs that left, but this difference is not statistically significant. We agree it is necessary to clarify this, so we added this information to the paper.

Fifth, what are the eight positions on hockey? Hockey teams typically have six players on the ice - a goalie, two/three defenders and two/three forwards. If you distinguish four positions in football, then three is certainly enough for hockey.

We are sorry for the misunderstanding. We distinguished between goalkeepers, left defense, right defense, left forward, and right forward. However, the large majority was categorized either as defensive or offensive. Additionally, some players (0.25% in Russia and 2.90% in Ukraine) were categorized for both offensive and defensive positions. Now, we keep the following groups: goalkeepers, defenders, forwards, and undecided (we update both regression and summary statistics).

Sixth, I simply cannot believe that over a period of seven seasons there were 54 different clubs playing in the Russian and 53 in the Ukrainian football league (this is what the numbers in your decriptive table seem to suggest).

We are thankful for this attentive comment. Indeed, this was not correct. We double-checked both tables and made sure that the summary statistics are correct.

Seventh, to what extent does the disappearance of Russian players from the Ukranian football league affect your estimations? Have you checked the robustness of your findings?

Eight, your map seems to suggest that Crimea hosts two clubs only. Are these both football or hockey clubs? What happens to your results if you estimate your model with/without these two clubs or include a dummy to distinguish these clubs from the rest?

This two issues are interrelated. First, there are fewer Russians in Ukraine after the crisis, but we control for the nationality both before and after the crisis, so we believe our results are robust. We can’t exclude Russians from the sample to test the robustness as they are the main variable of the interest. However, as Figure 3 didn’t include players from Crimea, it was misleading. We have now corrected the Figure and its description.

Second, thank you for the suggestion of such a robustness check. We have done the regressions you suggested. We understand that Russian players now play only in Crimea clubs (we didn’t report the results here just because without Crimea our variable of interest is omitted, so there is nothing to report). However, they’re discriminated. This is finding seems to be interesting, and we added the discussion of this in the paper (p.).

Finally, although not a native speaker myself, I find the English rather poor and the paper poorly edited.

We understand that some expressions we used can be confusing or misleading. Therefore, we sent our work to a proofreader. Attached you can find the certification.

**Reviewer #2:**

Summary
The authors of this paper set up an interesting natural experiment in which they use sport as a setting to test for discrimination in the context of recent geopolitical strife in the area. Overall, I think there could be some use from this study and interesting implications. However, the paper requires considerable editing and is a bit clunky throughout. I detail my concerns with that below. There is also some question as to whether or not the effect of interest is truly being identified. Based on my readings of the models, I am not confident that the discrimination effect is the primary way one could explain the results at hand. I think some additional robustness checks are necessary, and possibly some econometric specifications changed to do so. I detail my concerns in my comments below.

Dear reviewer, thank you for the time and effort that you put into improving this paper. We highly appreciate it. We tried our best to incorporate all the changes, also in accordance with the demands from the other reviewer. Please find detailed answers to the specific comments below:

Introduction and Background

The introduction is a bit disjointed and weaves back and forth between rote literature review and specifics about the results of the current study. This could use some work such that the literature frames the problem at hand and the hypotheses of the authors. One example is on Page 4, where the results of the study are discussed, then there is just some discussion of Becker’s theory of discrimination. It continues on with some information about Levitt’s discussion of economic discrimination, but doesn’t really tie the disagreement/difficulty directly to what this paper does (is it supposed to disentangle these two theoretical approaches? If so, it doesn’t give any indication that it does). And much of this seems out of order. Further, on Page 2, last paragraph, there is a list of papers that find influence on perception of groups, but this is not well tied into the current paper or used to frame any hypotheses. Some work on integration here is needed.

We are very thankful for this input. We restructure the introduction and create a literature review section (after the introduction). While we discuss the theory from both Becker and Levitt, we argue that taste-based discrimination is more appropriate for the current research and provide a brief explanation.

Additionally, we provide a concise overview of migration studies pertinent to the topic. First, in relation to sports in general, then regarding both Ukraine and Russia, and finally regarding a specific case that has similarities with our research.

Empirical Strategy and Estimations

-Substantially more needs to be provided about the DiD approach, assumptions behind this identification strategy, and so on. Most glaringly, it is unclear what the supposed control is: you are looking at the difference in the change of play time among Ukranian players in Russia (and/or Russian players in Ukraine) as compared to what, in particular? There is a pre-post period that is obvious, but you should be explicit about the comparison group (specifically: is it all players, just Russian players in Russia and Ukranian players in Ukraine, etc?)

We agree that this part was not comprehensive enough. We added a more detailed explanation of the control group’s formation:

“The difference-in-differences approach assumes that without the treatment, the treated group would show a trend similar to that observed for the control group (Villa, 2016). The choice of control group is crucial. To check the robustness of the results, we conduct the analysis using two options for control groups; We use as a control group all players in the corresponding league except the treated ones. Second, we use as a control group only foreign players in the corresponding league except the treated ones. To test the effect of the Crimean crisis on Ukrainians in the Russian league, the control group consists of all foreign players in the league except Ukrainians (and vice versa). The second control group formation assumes similar trends in participation and behaviour for foreign players in the league, whereas for domestic players the trend can be different.”

-Are minutes played possibly impacted by changes to the makeup of worker nationalities during this time? Specifically, fewer workers in Ukraine (or vice versa) could be a result of various things related to political conflict, including wanting (needing) to be closer to home, less economic ties across borders resulting in labor movement and concentration back home, etc. If this shift away from playing/working in a certain country is inherent in the minutes played, then you may be estimating something other than discrimination. The same goes for games played. Indeed, Figure 3 seems to show this is the case: nearly no Russians remained in the Ukranian soccer league.

We agree that the political conflict might affect the number of Ukrainians in Russia (or vice versa). This could affect our results. We agree that ideally there should be no roster change before and after the treatment, which is not our case, unfortunately. We believe that since we use individual-level data we observe those players who decided to stay or were forced to stay by their contract. The decision to stay on the team was already made, and our regressions estimate if their playing time is reduced. This is an important point, and we extend the discussion of this in the paper (p.12).

-If you are not including fixed effects for players, you may also be overestimating the effect by including changes to the makeup of nationalities in each country in your measurement of participation (i.e. minutes played goes to zero in Ukraine for a Russian player if that player moves to a team in Ukraine). This should at least be discussed to assuage concerns from readers about this issue. Although this could indicate hiring discrimination, there is little way to identify the causal effect here (as there are many other things that would seem likely to cause labor movement during geopolitical strife).

We have tried to estimate the models with player fixed effects. Almost none of the variables are statistically significant in such models, since we observe a significant portion of the players for small number of seasons. The player effects captures all the variation. Still, we believe we have a better indicator of player talent/quality than fixed effects. Our model includes transfermarkt.de evaluations of player value. This is a crowd-sourced metric of player market value. The advantage of this variable compared to fixed effects is that it varies from season to season. Such metrics are often used to control for individual or team skill (Bryson, Frick, and Simmons 2013; Frick 2007; Peeters 2018; Prockl and Frick 2018).

We agree that many things could likely cause labor movement during geopolitical strife, but since we analyze and observe those players who decided to stay in Russia/Ukraine, they already made their decision. Their player time is mostly affected by coaches’ decisions, which might be affected by his personal or fan attitude to the player. We have added this discussion to the text.

-You note that there is a UKR*crisis interaction, but there is no mention of a RUS*crisis interaction. This seems important, as an effect could be underestimated by only looking at, say, reduced play time for Ukranian players in a Russian league. If that play time is increasing Russian player play time (rather than just going to players of some other nationality), then the effect would be the combination of reduced Ukraine player play time AND increased Russian player play time in the Russian league, for example.

Assuming the presence of such effect, we made estimations without domestic players in a control group (models 1 and 2 in Table 3).

Additionally, we estimated the models with the control group including both domestic players and foreigners with additional interactions. The results are presented below. The interaction term for Russian players in the Russian league after crisis is negative, but not significant. The interaction term for Ukrainian players in the Ukrainian league after crisis is positive, but not significant, and small in magnitude.

|  | (1) | (2) |
| --- | --- | --- |
|  | rus_r2 | ukr_r2 |
| VARIABLES | log_av_min | log_av_min |
|  |  |  |
| Participated all games | 0.907*** | 0.948*** |
|  | (0.123) | (0.088) |
| Market value in euro | 0.081*** | 0.106*** |
|  | (0.011) | (0.018) |
| Age | 0.608*** | 0.339*** |
|  | (0.087) | (0.065) |
| Age sq. | -0.010*** | -0.006*** |
|  | (0.002) | (0.001) |
| Ukrainian after crisis | -0.555 | 0.057 |
|  | (0.351) | (0.118) |
| Russian after crisis | -0.043 | -0.449 |
|  | (0.089) | (0.326) |
| After crisis | 0.028 | 0.202 |
|  | (0.136) | (0.145) |
| Russian player | 0.100* | -0.165 |
|  | (0.057) | (0.148) |
| Ukrainian player | 0.210 | 0.069 |
|  | (0.190) | (0.059) |
| Zero market value | -1.031*** | -1.172*** |
|  | (0.152) | (0.106) |
| changed_team | -0.143 | 0.069 |
|  | (0.092) | (0.070) |
| Position dummies | Included | Included |
|  |  |  |
| Season dummies | Included | Included |
|  |  |  |
| Club dummies | Included | Included |
|  |  |  |
| Constant | -5.118*** | -2.353** |
|  | (1.184) | (0.888) |
|  |  |  |
| Observations | 2,935 | 2,770 |
| R-squared | 0.253 | 0.269 |

Robust standard errors in parentheses

*** p<0.01, ** p<0.05, * p<0.1

Results and Discussion

-It would be useful to flag statistical significance in Table 3, 4, 5, and 6. Also should include a note as to what is in the parentheses under each coefficient estimate (I am assuming it is the standard errors, but it is not clear).

This is done now, it was our mistake not to do it.

-When you note the marginal effect is 52% (Page 14), please put this in language that relates to the units your effect is reported in (52% decline in minutes played? That seems like an enormous effect – is this believable?). I want to be sure I’m interpreting this correctly.

This is 52% of average playing time. The size of the effect is robust to exclusion of distance to Crimea. As you see below (the answer to the next comment), in the regression the coefficient is -62, which gives marginal effect of (exp(-0.62)-1)*100 = -46.2% considering that dependent variable is in logs. We have added the broader discussion of this result to the paper.

-It would be good to see an average discrimination effect without the inclusion of the miles away from Crimea coefficient estimate. Sometimes interpreting these with all the additional interactions can get confusing. This should be easy to do. This might be particularly important in the context of Table 4, where there are some competing effects of the UKR*crisis and the UKR*crisis*Distance interactions.

Thank you for this suggestion. We have these results (see Table 7 in the section “online appendix”, p 32). We decided not to include this to the summation initially, since the major result is in line with the results presented in Table 3. We agree that it is easier to interpret the marginal effect without continuous interaction, so we have added this discussion to the paper. As you see, the significance and the size of the effects of the main variables of interest are robust to exclusion/inclusion of distance to Crimea. We have added this regression to the appendix.

-Are penalty minutes measuring discrimination by referees or increased aggression among Ukranian players, given the geopolitical situation? This would be hard to disentangle; however, it should at least be addressed by the authors as a possible confound.

We agree. We have added this discussion:

“In Table 6, we change the dependent variable to the penalty minutes received. The results show that Ukrainians received statistically significantly more penalty minutes after the crisis. It is important to note that penalty minutes might either be the result of player discrimination by referees or increased aggression among Ukrainian players, given the geopolitical situation. However, the data doesn't allow us to distinguish these two effects.”

The authors note “there is no evidence that Russian hockey players in Ukraine behavior more violently…” but it is not clear how the authors came to this conclusion (and it is about Russian players, rather than Ukrainian players – so I am confused here).

We have corrected this paragraph and now refer to models while describing the results.

The discussion, as with the introduction, needs some major organization fixed and some tightening up in the transitions so the reader does not get confused what model(s) you’re referring to from sentence to sentence.

We agree that several parts of both discussion and conclusion were not clear. We restructured the introduction and several parts of the conclusion. Because referring to the tables was confusing, we started the respective paragraphs with explanations of the table and then the models. We repeated this process for every table. To improve readability, we specifically mention each dependent variable on top of each table.

Finally, a proofreader corrected the article as reviewer 1 mentioned that the English language was not sufficient. Thus, several expressions changed.
