# Supplementary material for: After the Crimea crisis: Employee discrimination in Russia and Ukraine
Source: PLoS One. 2020 Oct 28;15(10):e0240811. doi: 10.1371/journal.pone.0240811 (PMC7592748; doi:10.1371/journal.pone.0240811)
Supplement: Attachment — Submitted filename: Reviewer.docx [file pone.0240811.s003.docx]

Reviewer #2: I appreciate the authors addressing many comments in turn in my previous review. However, I am having difficulty finding the magnitude of the effect in the paper to be believable without additional evidence and explanation. I lay out remaining concerns below and note why I think the econometric model is not properly specified:

*Thank you for your comments. We have tried to address the comments. We have added the discussion of the effect size and the description our robustness checks to the paper.*

1) Given the acknowledgement that it is not possible to distinguish between player behavior resulting in more cards vs. bias and discrimination causing more cards, the contribution of this portion of the analysis seems rather low. Given the length of the empirical analysis – and need for more clarification on the models – I would suggest focusing on the play time variables alone, and removing the yellow card analysis.

*We agree with the reviewer. We exclude the analysis from the paper but add a footnote (page 2).*

2) The taste based theory is being used to drive the question; however, the authors need to extend this into the “minutes played” portion of the analysis. As is, the way it is described refers to hiring. The authors ignore all changes to employment, and only look at minutes. This can present a disconnect for readers less familiar with this economic approach to discrimination.

*We agree with the reviewer that we did not want to focus on the hiring process but on the situation when a player is already hired. We update the description in the literature section and include an explanation regarding Beckers taste based discrimination theory in the econometric strategy sector (when we mention “minutes” for the first time).*

3) The other issue that comes up with Becker’s theory here is that if the purported discrimination takes place across the board, then this does not put anyone at a disadvantage. The inability for markets to drive out discrimination has long been a criticism of the Beckerian approach. Are teams that play even fewer foreign players actually worse off?

*This is an interesting topic. Research does not give a clear response. Some research shows that diversity is positive in the sports environment (up to some extent). For example, Bell and Riol (2017) examine college basketball and find a positive effect on communication. Prinz and Wicker (2016) examine how team diversity influences team performance. They find evidence in favor and against diversity. Szymanski (2017) examines how biculturalism influences national association football. The results are in favor of diversity. However, Maderer et al. (2014) examine team football and find exclusively negative effects on performance. We add a brief description in the text.*

*Bell, R., & Riol, C. F. (2017). The impact of cross-cultural communication on collective efficacy in NCAA basketball teams. International Journal of Cross Cultural Management, 17(2), 175-195.*

*Maderer, D., Holtbrügge, D., & Schuster, T. (2014). Professional football squads as multicultural teams: Cultural diversity, intercultural experience, and team performance. International Journal of Cross Cultural Management, 14(2), 215-238.*

*Prinz, J., & Wicker, P. (2016). Diversity effects on team performance in the Tour de France. Team Performance Management.*

*Szymanski, M. (2017). Team effects of bicultural individuals: insights from football team performance (Doctoral dissertation).*

4) It is not clear why Russia seeing annexation as a “natural process” is in any way counter to either discrimination or aggressive behavior. The authors need to explain their reasoning here more.

*We agree that this explanation was not clear enough. We rephrased the paragraph and explain the reasoning in more detail.*

5) Make sure to be clear that you restrict sample to players appearing before and after. This is still not completely clear in the methods.

*We are sorry for the misunderstanding. The sample restriction for players appearing before and after was only an additional robustness check. The results presented in the paper are based on the data for all players.*

6) The choice of random effects over fixed effects for players being justified by whether the coefficients of interest are statistically significant is seriously troublesome. Did you run a Hausman test to defined your random effects choice? I have a strong feeling this is what’s driving the enormous effect size. You’re likely attributing differences in play time across players to differences within player, and claiming this is the size of the effect. If the authors think FEs for players overly control for things they seek to estimate, they need a very convincing case as to why and what the structure of that choice is. As it is, there is not a convincing case (the transfrmarkt data is useful as a control, but it’s then not clear why the effect size would change so much with FEs in lieu of this labor valuation data).

*We do not use random effects. We revised the methodology section to make it clear (p.7, highlighted in yellow). Instead, we included club effects and transfermarkt players value. As a substantial portion of players stayed for a small number of seasons, usage of fixed effects for players results in a boost of the standard errors.*

7) The effect size is just not believable and is in the range of 10 or more times the magnitude that other studies of discrimination like this have found. And given that the authors ignore dropouts (restricting to players observed only before/after), this would suggest their estimate of discrimination is biased downward. The combination of the huge effect size and the likely downward bias of the effect size tells me something is amiss in the data and analysis, there are a few overly influential players driving the effect, or the authors are incorrectly applying between player differences in play time to changes within player.

*Thank you for this comment and your suggestions. We agree that the effect is high in comparison with previous studies. However, the most relevant studies consider racial discrimination or discrimination against migrants. In our case, relations between two countries deteriorated sharply and rapidly, that resulted in much stronger effect. It is even described as a “war” in the literature, see, for example, the following studies:*

*Shevko, Demian; Khrul, Kristina (2017). "Why the Conflict Between Russia and Ukraine Is a Hybrid Aggression Against the West and Nothing Else". In Gutsul, Nazarii; Khrul, Kristina (eds.). Multicultural Societies and their Threats: Real, Hybrid and Media Wars in Eastern and South-Eastern Europe. Zürich: LIT Verlag Münster. p. 100. ISBN 9783643908254*

*Snyder, Timothy (2018). The Road to Unfreedom: Russia, Europe, America. New York: Tim Duggan Books. p. 197. ISBN 9780525574477. Almost everyone lost the Russo-Ukrainian war: Russia, Ukraine, the EU, the United States. The only winner was China.; Mulford, Joshua P. (2016). "Non-State Actors in the Russo-Ukrainian War". Connections. 15 (2): 89–107. doi:10.11610/Connections.15.2.07. ISSN 1812-1098. JSTOR 26326442.*

*Kofman, Michael; Migacheva, Katya; Nichiporuk, Brian; Radin, Andrew; Tkacheva, Olesya; Oberholtzer, Jenny (2017). Lessons from Russia's Operations in Crimea and Eastern Ukraine (PDF) (Report). Santa Monica: RAND Corporation. p. 69.*

*As we noted above, the sample is not limited to the players observed only before/after (we have updated the description of regression analysis).*

*To test whether our results are robust to outliers, we conducted an additional analysis winsorizing the continuous variables (average minutes played, transfermarkt player value, and age) at the 1st and 99^th^ percentiles (model 2) and at the 2^nd^ and 98^th^ percentiles (model 3).*

*The results for the Russian soccer league are presented below. Model 1 is our basic model, presented in the paper. The effect is robust, it decreases slightly in magnitude. For the Ukrainian soccer league, the results are also robust with the magnitude of the effect increasing slightly and staying at the same 10% significance level.*

|  | (1) | (2) | (3) |
| --- | --- | --- | --- |
|  | **BASIC** | **1 & 99 percentiles winsorization** | **2 & 98 percentiles winsorization** |
| VARIABLES | Dependent variable: log(average minutes | Dependent variable: log(average minutes | Dependent variable: log(average minutes |
|  |  |  |  |
| **Ukrainian after crisis** | **-0.620*** | **-0.600*** | **-0.572'** |
|  | **(0.331)** | **(0.339)** | **(0.344)** |
| After crisis | 0.277* | 0.257* | 0.258* |
|  | (0.139) | (0.141) | (0.142) |
| Participated all games | 0.745*** | 0.675*** | 0.653** |
|  | (0.188) | (0.237) | (0.233) |
| Market value in euro | 0.062*** | 0.094*** | 0.101*** |
|  | (0.008) | (0.010) | (0.012) |
| Age | 0.218** | 0.137 | 0.110 |
|  | (0.095) | (0.099) | (0.103) |
| Age sq. | -0.003* | -0.002 | -0.001 |
|  | (0.002) | (0.002) | (0.002) |
| Ukrainian player | 0.152 | 0.152 | 0.141 |
|  | (0.182) | (0.190) | (0.194) |
| Zero market value | -1.239*** | -1.260*** | -1.229*** |
|  | (0.261) | (0.259) | (0.253) |
| Team change during season | 0.142 | 0.134 | 0.131 |
|  | (0.120) | (0.116) | (0.114) |
| Player position effects | included | Included | Included |
| Club effects | included | included | Included |
| Season effects | included | included | included |
| Club Position | -0.060** | -0.059** | -0.058** |
|  | (0.027) | (0.027) | (0.027) |
| Constant | 0.269 | 1.358 | 1.701 |
|  | (1.312) | (1.325) | (1.343) |
|  |  |  |  |
| Observations | 1,268 | 1,268 | 1,268 |
| R-squared | 0.201 | 0.210 | 0.212 |

Robust standard errors in parentheses

*** p<0.01, ** p<0.05, * p<0.1, ' p<0.15

If players are losing *half* their playtime, this would not go unnoticed and there would not need to be a regression to find that out. Given that, if there is a useful story or specific examples in the news that can help clearly identify this happening, then the authors should refer to this. If there is not, then there needs to be a serious explanation as to how and why we would expect to see an effect this large go completely unnoticed. A claim this extraordinary needs some supporting evidence, and an effect size this large would be easily picked up by any spectators or media covering games.

*We agree with the reviewer. The effect, however, is not unnoticed in the media. There are articles about dramatic change in fans’, authorities’, and other players’ attitudes towards Ukrainian and Russian football players after the Crimean crisis. For example, discussion of pressure on Ukrainian soccer players in Russia* (Sansun, 2018)*, attitude of Ukrainian players towards Russia* (*"Russia Attacked*, 2020)*, or analysis of reaction to the Ukrainian soccer plaeyer Rakitsk) transfer to Russian top club* (Wesolowsky, 2019)*.*

*“Russia attacked:” Ukrainian football player explains why he won’t play for Russians. (2020, May 4). Unian.Info. https://www.unian.info/politics/ukrainian-football-member-of-national-team-explains-why-he-won-t-play-for-russia-10982465.html*

*Sansun, D. (2018, March 17). Ukrainian Footballers in Russia Could Face Uncertain Future. Russian Football News. http://russianfootballnews.com/ready-ukrainian-footballers-in-russia-could-face-uncertain-future/*

*Wesolowsky, T. (2019, January 31). Kick In The Grass: Fans Jeer Ukrainian Soccer Player’s Transfer To Russia. RadioFreeEurope/RadioLiberty. https://www.rferl.org/a/fans-jeer-ukrainian-soccer-player-s-transfer-to-russia-rakitskyy/29744161.html*

*We have added the description of the conflict, and added the discussion of the effect size and our robustness checks.*

It might also be useful to know if these are “stars” or if they’re largely low tier players with the purported discrimination in play time happening at the margin of bench players (where quality differences are not large). The latter would go more easily undetected. But the size of this effect really needs to be addressed and discussed, and I suspect it has more to do with the econometric specification than anything else.

*Thank you for this comment. To test this, we included an interaction term with a dummy on a star player. We defined a star player as a player with a transfermarkt value higher than 1 million euros (model 4) or 2 million euros (model 5). Model 1 is our basic model, presented in the paper. The results show even higher discrimination than in the basic model. The interaction, however, is not significant. The result is robust to the threshold for star player definition. Thus, there is no evidence that the Crimean crisis had a different effect on star players and non-star players in Russia. We are unable to test this for the Ukrainian league as there are no Russian star players both before and after 2014 in our sample.*

|  | (1) | (4) | (5) |
| --- | --- | --- | --- |
|  | **BASIC** | **Star Player > 1 mln euros** | **Star Player > 2 mln euros** |
| VARIABLES | Dependent variable: log(average minutes | Dependent variable: log(average minutes | Dependent variable: log(average minutes |
|  |  |  |  |
| **Ukrainian after crisis** | **-0.620*** | **-0.833*** | **-0.670*** |
|  | **(0.331)** | **(0.463)** | **(0.359)** |
| **Ukrainian after crisis · Star player** |  | **0.555** | **0.408** |
|  |  | **(0.543)** | **(0.613)** |
| Participated all games | 0.745*** | 0.731*** | 0.758*** |
|  | (0.188) | (0.177) | (0.198) |
| Market value in euro | 0.062*** | 0.054*** | 0.044*** |
|  | (0.008) | (0.007) | (0.007) |
| Age | 0.218** | 0.138 | 0.154* |
|  | (0.095) | (0.088) | (0.083) |
| Age sq. | -0.003* | -0.002 | -0.002 |
|  | (0.002) | (0.002) | (0.002) |
| Star player |  | 0.238** | 0.363*** |
|  |  | (0.096) | (0.106) |
| After crisis | -0.018 | 0.263* | 0.264* |
|  | (0.151) | (0.146) | (0.148) |
| Ukrainian player | 0.152 | 0.147 | 0.138 |
|  | (0.182) | (0.196) | (0.198) |
| Zero market value | -1.239*** | -1.198*** | -1.230*** |
|  | (0.261) | (0.265) | (0.265) |
| Team change during season | 0.142 | 0.141 | 0.142 |
|  | (0.120) | (0.117) | (0.116) |
| Player position effects | included | Included | Included |
| Club effects | included | included | included |
| Season effects | included | included | included |
| Club Position | -0.060** | -0.059** | -0.058* |
|  | (0.027) | (0.028) | (0.028) |
| Constant | 0.269 | 1.269 | 1.102 |
|  | (1.312) | (1.124) | (1.110) |
|  |  |  |  |
| Observations | 1,268 | 1,268 | 1,268 |
| R-squared | 0.201 | 0.207 | 0.209 |

Robust standard errors in parentheses

*** p<0.01, ** p<0.05, * p<0.1

8) It would be worth rescaling your distance from Crimea variable so that your coefficient is not 0.000.
*Thank you for this comment. We corrected this.*

9) It is not clear to me why the Distance to Crimea variable itself would have any significant coefficient. Are teams and their players (all of them) further from Crimea just playing more minutes overall? Otherwise, this seems like a strange result in your data.

*The effect is negative, so the average player of northern team plays less minutes. We agree that this seems strange. As we capture time-constant club characteristics with club-level dummies, it seems that there is something, time-variant, which is correlated with the distance from Crimea. We do not have clear explanation for this. Distance from Crimea is not correlated with club total market value, which is a proxy of budget. We suppose that distance from Crimea might capture climate characteristics of the host cities of football clubs. Northern clubs might have bigger roster to vary players during the season. We have added this description to the text.*

10) To be able to see these possible effects, I think the authors need to cross-tabulate them in their summary tables. In other words: show minutes played by nationality (Russian or Ukranian) specifically for each league, on average. Then also include this before/after the crisis. I think looking at the transfrmarkt value crosstabulated in the same way would be enlightening to the reader to know what kinds of players are represented in your treated sample.

*Thank you for this comment. Below there is a table with cross-tabulated minutes played per game by the league, player nationality, and political crisis. In the Russian league, players of all nationalities started to play slightly more on average after the crisis. This is true for Ukrainian players as well. In the Ukrainian league, players of all nationalities play less after the crisis, however, there is a substantial decrease in time played per game for Russian players.*

*Table. Cross-tabulated minutes played per game*

| League | Player nationality | | |  |
| --- | --- | --- | --- | --- |
|  |  | | |  |
|  | Russian | Ukrainian | Other | |
|  |  |  |  | |
| Russia |  |  |  | |
| Before crisis | 22.361 | 18.138 | 28.659 | |
| After crisis | 23.518 | 23.974 | 34.032 | |
|  |  |  |  | |
|  |  |  |  | |
| Ukraine |  |  |  | |
| Before crisis | 21.533 | 30.053 | 34.295 | |
| After crisis | 5.774 | 28.463 | 33.711 | |

*The cross-tabulation of the average transfermarkt value of players shows that in the Russian league the quality of the players has not changed significantly. On average, Ukrainian players had a higher value than Russian ones both before and after 2014. In the Ukrainian league, the situation is the opposite. Even before the crisis, Russian players on average had a lower value and it became even lower after the crisis. However, in the regression analysis, we control for player’s transfermarkt value to capture the change in the minutes played due to the difference in player’s quality.*

*Table. Cross-tabulated average transfermarkt value of players, mln euros*

| League | Player nationality | | |  |
| --- | --- | --- | --- | --- |
|  | Russian | Ukrainian | Other | |
|  |  |  |  | |
| Russia |  |  |  | |
| Before crisis | 1.139 | 1.446 | 2.782 | |
| After crisis | 1.118 | 1.306 | 2.938 | |
|  |  |  |  | |
|  |  |  |  | |
| Ukraine |  |  |  | |
| Before crisis | .308 | .709 | 1.825 | |
| After crisis | .09 | .610 | 2.360 | |

11) Also, in Table 1, you note that of 2,935 player-year observations, only 2% in Russia are Ukrainian and same for Russian players in Ukraine. If your n = 2,935 are correct, you are making inferences based on only 8 players in each league. This relates to my point in comment #6 above regarding an outsized effect of a very, very small number of observations that are actually treated in the model (nearly none of the observations fall into the treatment group).

*We are sorry for the misunderstanding. As we mentioned above, our sample includes all players, not only those who appeared both before and after 2014. Particularly, for the Russian league, we have 32 Ukrainian players and 51 observations as most of them played 1-2 seasons. Moreover, in the regression models with only foreign players as a control group, the number of observations is lower - 1,268 for the Russian league. Thus, in these models the percentage of Ukrainian players is 4%. We added a brief note in the Empirical strategy and estimations subsection about the percentage of treatment group.*
